# The influence of visual marketing on consumers' purchase intention of fast fashion brands in China–An exploration based on fsQCA method

**DOI:** 10.3389/fpsyg.2024.1190571

**Published:** 2024-04-08

**Authors:** Yaqiong Zhang, Shiyu Huang

**Affiliations:** Business School, China University of Political Science and Law, Beijing, China

**Keywords:** fast fashion brands, visual marketing psychology, consumers' purchase intention, China, fsQCA

## Abstract

Under the rapid development of e-commerce, offline brick-and-mortar stores have been severely impacted. However, the importance of the visual, sensory and even psychological experience in the apparel industry makes offline stores still irreplaceable. The impact on consumers' visual experience cannot be ignored and is a significant influencing factor in determining consumers' psychological change and purchase intention. Especially for fast fashion brands which pursue low costs, visual marketing strategies is a cost-effective marketing tool to enhance the visual experience. In this paper, by adapting SOR theory and using fuzzy set qualitative comparative analysis (fsQCA) research method, 15 fast fashion apparel brands and 374 valid questionnaires are adapted in China to explore not only the influence of individual dimensions in visual marketing on consumers' purchase intention, but also the action of multi-dimensional combinations. The research finds that: (1) there are two driving paths for high consumers' purchase intention. The first path is a combination of high clarity of arrangement and low display density; the second path is a combination of low light intensity, high clarity of arrangement, high tonal harmony and high window appeal. (2) There are also two paths that drive non-high consumers' purchase intentions, and they are asymmetrically related to the paths that drive high consumers' purchase intentions. The findings of this study help to provide direction and suggestions for offline visual marketing strategies of fast fashion apparel brands to increase consumers' psychological perception and purchase intention through a range of visual presentation techniques.

## 1 Introduction

According to the China Business Industry Research Institute (2021), because of the massive growth of local apparel brands and the influx of foreign brands, China has become the world's largest apparel market. The total sales volume of China's apparel market reached 1.932 trillion yuan in 2019, with a year-on-year growth rate of 7.8%; by 2020, the market has reached 1.342 trillion yuan (China Business Industry Research Institute, 2021). In this highly competitive environment, apparel companies must continuously improve its competitive advantage in order to survive and gain consumers' recognition (Ye et al., 2023). Moreover, in an era of rapid development of e-commerce in China, the apparel industry in China has also undergone radical changes (Zor, 2023). A growing number of consumers are turning from offline shopping to online platform channels to select and purchase products (Khwaja et al., 2022; Timoumi et al., 2022). The online apparel shops quickly crowding out offline stores by virtue of their convenience, speed and low cost (Liu et al., 2019; Yeo et al., 2022). However, unlike other fast-moving consumer goods industries, the apparel industry is somewhat unique in that consumers rely more on the real shopping experience, environmental experience and fitting services that can be offered in offline brick-and-mortar shops (Falode et al., 2016; Orús et al., 2021). As a result, many apparel brands try to retain offline consumers by capturing the characteristics of consumers who value experience and adopting a variety of relevant marketing tools (Liu et al., 2019; Yeo et al., 2022). Data from McKinsey.com (2021) shows that in the apparel category, the number of products purchased by consumers in online channels accounts for 21%−50% of overall consumption, with a larger proportion of consumers still willing to purchase products through offline shops. This shows that offline stores of apparel brands are still an important way for consumers to occur their purchasing behavior and are difficult to be completely replaced by online shops.

Because of the globalization and the Internet development, many famous foreign clothing brands have entered the Chinese market (Su, 2016; Furr et al., 2022). The common features of these brands are fast updates, low inventory, low prices and following fashion trends, collectively known as fast fashion brands, which are the pioneers of the apparel industry today (Shen et al., 2017; Wei and Jung, 2022). Domestic apparel brands have followed suit, creating a situation in which fast fashion apparel brands are blossoming (Su, 2016; Furr et al., 2022). According to data from the China Fast Fashion Apparel Industry Research and Future Prospects Forecast Report, a total of 356 new shops were added by fast fashion brands to the market in China in 2020, an increase of 19% over the same period in 2019 (China Fast Fashion Clothing Industry Research Future, 2021).

Due to the characteristics of fast fashion brands, such as “quick release, low price, low inventory and following the trend,” in order to reduce costs and losses, companies are unable to spend as much on advertising as other types of apparel industry, they usually enhance the experience of offline shops to attract consumers through visual marketing (Kleih and Sparke, 2021). Visual marketing is a method of marketing technology, but also a visual experience; it refers to the use of multiple dimensions of vision to achieve the purpose of selling products or brand promotion, such as: window demonstration, display layout, lighting effects (Sevilla and Townsend, 2016; Hussain, 2019; Chae et al., 2021; Kleih and Sparke, 2021). In addition, visual marketing approach is relatively inexpensive and effective. By using visual marketing strategy as a cost-effective marketing method, fast fashion brands can significantly reduce the price of their products and meet the needs of their target customers for fashionable clothing at low prices (Liu et al., 2019; Chae et al., 2021). In addition, the combination of the 'fast on-trend' nature of fast fashion brands and good visual displays can create a synergy that can increase consumers' purchase intention (Huang et al., 2020). However, inappropriate and boring visual images of offline shops can lead to boredom and aesthetic fatigue among consumers (Huang et al., 2020). To sum up, due to the characteristics of the apparel industry and the importance of visual and sensory experience in the fast fashion industry, fully utilizing the advantages of offline visual marketing can attract consumers, further impact and determine consumers' purchase intention, and is a significant way to stand out in the fast fashion apparel brands. On the other hand, the positioning of the fast fashion apparel brands makes brands more inclined to choose low-cost visual marketing methods such as using offline shop layouts and displays.

Through a systematic review of the relevant literature, this study found that most of the existing research focus on exploring single dimension of visual marketing rather than a configuration perspective to investigate the influence of visual marketing on consumers' purchase intention (Moore and Zhang, 2015; Liu et al., 2019; Huang et al., 2020; John and De'Villiers, 2020; Sample et al., 2020; Pauluzzo and Mason, 2022). However, the impact of visual marketing strategy on consumers' purchase intentions is a combination of multiple dimensions, and focusing on a single dimension alone does not provide a comprehensive and in-depth picture of the relationship between the two variables. Therefore, how to provide an enjoyable visual experience in offline stores through a comprehensive visual marketing strategy, so that consumers can feel the pleasant and relaxing shopping atmosphere, immerse themselves in a visual feast, feel the charm of the products, and thus increase their purchase intention is vital for fast fashion industry. Therefore, based on the theory of SOR, this paper aims to investigate not only the influence of individual dimensions in visual marketing on consumers' purchase intentions, but also the mechanism of action of multi-dimensional combinations. In order to achieve the purpose, a fuzzy set qualitative comparative analysis (fsQCA) research method has been used and 15 fast-fashion apparel brands has been chosen as research objects. This paper not only enriches the existing theory and literature related to visual marketing and consumers' purchase intention, but also provides effective marketing strategies for fast fashion industry in a competitive market environment. Especially for fast fashion apparel brands pursuing low cost, the full use of visual marketing strategies to enhance the visual experience in stores is a cost-effective and effective marketing tool.

## 2 Literature review

### 2.1 Visual marketing

Visual marketing is a method of marketing technology, but also a visual experience; it refers to the purpose of product marketing or brand promotion through visual (Zhang and Zhang, 2023). Visual marketing includes the following: Spatial: creating a brand atmosphere through spatial three-dimensional visual effects. Graphic: through graphic visuals and posters, etc. as a visual effect. Media: to express the concept of visual merchandising through promotional forms. Display: to complete the internal structural changes. Styling: to complete the optimization and integration of the image. Through a series of visual communication to express the concept of visual marketing and the core part (Yu et al., 2018). There is a wealth of research on visual marketing, and most of the existing research is concerned with the impact of visual marketing on consumer behavior and psychology. The origin of the concept of “visual marketing” can be traced back to the 1970s and 1980s in the United States, initially as a part of the retail sales strategy. In Visual Merchandising Marketing (VMD) magazine, the National Retail Federation defines visual marketing as a method that presents products to the marketplace through a range of visual presentation techniques and tools. Visual marketing was first applied to the apparel industry by American designers, and as a result, the sales model for the apparel industry advanced dramatically (Huang et al., 2020). In the late 1990s, Schmidt and Simonson's research laid the theoretical foundations for visual marketing, which fully illustrated the positive effects of visual marketing on brand competitiveness and divided visual marketing into three dimensions: the product itself, the spatial structure and the mode of communication (Bockholdt et al., 2020). Based on previous research, Morgan and other scholars outlined the history of visual marketing and provided a comprehensive and unique analysis of visual displays both in-store and in windows, illustrating the importance of visual marketing to the consumer buying experience (Morgan et al., 2009; Steidle and Werth, 2014; Sevilla and Townsend, 2016; Hussain, 2019). In Moore and Zhang (2015) studied visual marketing strategies for apparel brands to attract the buying interest of consumers in the new era, illustrating the importance of visual marketing to the development of the apparel industry in the Internet era. In 2020, Sample et al. (2020) conducted an in-depth analysis of visual marketing cases in offline clothing shops from the perspective of consumer psychology, showing various display designs in windows and shops. Furthermore, John and De'Villiers (2020) analyzed how visual media impact consumers' purchase decisions. Focus on the domestic research on visual marketing, there are several relevant research related to the window, store display, spokesmen visual image and other dimensions (Liu et al., 2019; Gao et al., 2020). Furthermore, Huang Jing and others summed up the existing visual marketing achievements and sorted out the five most important dimensions: the interaction between vision and other senses, display, position, light, and shape, this paper provides a structural guide for the further research of visual marketing (Jadva et al., 2010; Steidle and Werth, 2014; Bertamini et al., 2016; Huang et al., 2020).

### 2.2 Fast fashion

The fast fashion industry is currently being studied in a variety of ways, including its origins and marketing characteristics and strategies (Cook and Yurchisin, 2017). Fast fashion, or “fast fashion,” is a new business model that has entered the history of the apparel industry, aiming to sell clothing containing the latest fashion elements to consumers as quickly as possible and at a low price (Ye et al., 2023). Compared to the traditional apparel industry, the fast fashion industry is fast, inexpensive and on-trend (Mrad et al., 2020). In addition, unlike the traditional marketing methods used by other clothing companies, fast fashion apparel brands focus more on visual marketing techniques such as matching displays, light and shop layouts to attract consumers (Rese et al., 2022).

Focusing on the origins and development of the fast fashion industry, Japanese scholar Ohmae (1996) analyses the background of the emergence of fast fashion - the “M-shaped society,” where the rich and the poor are severely divided and the number of people in both income brackets has increased, with the lower-income and middle-income groups preferring to buy lower-priced. However, the desire for luxury makes them want affordable clothing with the fashionable style of high-end brands. While fast fashion brands are less expensive and may be inferior in quality to other higher priced apparel brands, they are fashionable and updated quickly (Zhang and Zhang, 2020). As a result, fast fashion clothing brands have grown rapidly in recent decades by accurately capturing the psychology of consumers (Bhardwaj and Fairhurst, 2010). In addition, Barnes and Lea-Greenwood (2010) pointed out that fast fashion apparel brands target a similar audience group, mostly younger consumers aged 15–35, who have a strong desire for fashionable things but cannot afford the high price of clothing and do not have high requirements for the quality of clothing. However, the visual experience of offline shops of different brands varies greatly, which will also affect consumers' purchase intention and other consumer behavior of fast fashion apparel brands especially the young generation (Pauluzzo and Mason, 2022).

In addition, Barnes and Lea-Greenwood (2010) proposed ways for fast fashion brands to improve supply chain efficiency, with an emphasis on the importance of offline shop environments. Then, Cachon and Swinney (2011)conducted an in-depth analysis of the “quick response” characteristics of the fast fashion industry, and pointed out that the traditional way of displaying products according to product type was no longer suitable for consumer needs, and that fast fashion clothing displays should focus more on thematic design. Based on this research, scholars have begun to explore how visual elements can be better integrated with marketing methods to promote fast fashion brands. Based on the 4P theory, Liu and Zhang (2011) concluded the marketing characteristics of fast fashion brands, such as “More style, less quantity,” and pointed out that fast fashion brands mainly sell in offline stores, mostly in the core business circle. In 2012, Liu (2012) suggested that compared to the traditional apparel industry, the fashion cycle of fast fashion brands is shorter, generally only 4–4 years are shorter, typically only 4–6 weeks, which allows them to create more sales seasons. In addition, unlike traditional marketing methods such as heavy advertising used by other apparel companies, fast fashion apparel brands focus more on using cost-effective visual marketing techniques such as matching clothing displays and shop layouts to attract consumers' purchase intention (Mrad et al., 2020).

In terms of how to better attract consumers, Pantano et al. (2019) proposed that fast fashion brands manage their offline shop displays in a standardized way to give consumers a clear visual impression of their products, thus generating the perception that they are good value for money. Huang et al. (2012) proposed that fast fashion brands focus on consumer needs in the marketing process in order to attract consumers' interest in buying more efficiently and precisely without wasting money. Scholars generally believe that visual marketing has a driving effect on fast fashion apparel brands. Yogantari and Dwijendra (2020) proposed that fast fashion apparel brands have reduced the negative impact of discounting on brand value through the clever use of visual marketing strategies. On the other hand, Roozen and Raedts (2020) explored the impact of the visual media impact on the attitude of consumers toward fast fashion apparels in the fast fashion industry.

### 2.3 Consumers' purchase intention

Consumer purchase intention refers to the subjective probability or tendency of consumers to buy certain goods or services (Wang et al., 2023). In practical application, many scholars divide it into two dimensions: purchase intention and recommendation to others (Wang et al., 2023). Consumer demand can be guided, and can be enhanced by artificially creating external stimuli to generate, then grabbing the customer's eyeballs is a prerequisite for marketing success. Brands using effective visual design can better stimulate consumers' interest in purchasing, extend their stay in the store, and induce them to convert their potential shopping needs into actual purchasing behavior. Consumers' purchase intention is the initial judgment and subjective tendency of consumers before the purchase behavior, which can predict the purchase behavior of consumers more accurately (Gunawan and Huarng, 2015). It is an important precondition for the purchase behavior of consumers after they have certain knowledge of the product and evoke the desire to buy. The factors that influence consumer purchase intention are more diverse, and how to improve consumers' purchase intention through the analysis of purchase factors has been the concern of scholars, and there is a considerable amount of research on this topic. Specifically, consumer environment influences consumer sentiment and thus consumers' purchase intentions (Dash et al., 2021). Malone (2011) showed that the level of corporate social responsibility has an impact on consumers' purchase intentions, and that this impact is related to consumer personality traits. In addition, Madahi and Sukati (2012) suggested that consumers' purchase intentions are influenced by price, packaging, product quality and other factors. Followed it, Li M. et al. (2020) found that among the visual marketing dimensions, offline physical shops have the most direct impact on consumers' purchase intentions. Consumers not only pay attention to clothing items, but also to the visual information of the shops. Among them, the fitting rooms and window images of offline shops have a greater impact on consumers' purchase intention. In summary, consumers' purchase intention is influenced by their own characteristics, the product itself and the consumer environment. For the apparel industry, the visual environment of offline shops will greatly influence consumers' purchase intention.

### 2.4 Theory of S-O-R

The S-O-R theoretical model, i.e. Stimulus-Organism-Response, is one of the important theoretical models in the field of psychology (Alanadoly and Salem, 2022; Türkdemir et al., 2023). Regarding the S-O-R theory has more application areas, focusing on the field of consumer behavior research, the three variables of the S-O-R model represent S-External Stimulus (including external macro- and micro-market stimuli); O-Organism, which includes consumer's perceptual and affective variables; and R-Response variable (the consumer's behavior resulting from internal changes under the influence of external stimuli). Therefore, the S-O-R model can be summarized as the consumer's perception and emotion changes under the stimulation of the external environment, which results in behaviors such as using, purchasing, and recommending (Zhang et al., 2017; Liu et al., 2018; Luo and Lü, 2019; Li Q. et al., 2020; Huang et al., 2022).

With the rapid development of fast fashion apparel brands, the rise of visual marketing and the importance of physical apparel stores, consumers in the process of selecting and purchasing apparel, managers and operators of physical stores will describe and display the store and products through multi-dimensional visual marketing strategies, which will stimulate the consumers' perception of the marketing effect and promote consumers' purchasing behavior. Based on this, scholars have begun to apply the S-O-R model to study consumer shopping behavior, which describes the external stimuli S during the offline shopping process, including: a series of visual presentations such as the brand and corporate image, in-store lighting, colors and displays, etc.; the organismic changes O, including: perceived value (Liu et al., 2018; Han and Xu, 2019), perceived excitement (Han and Xu, 2019), perceived trust and perceived usefulness (Han and Xu, 2019; Zhang and Zhang, 2020), perceived quality and perceived risk (Zhao and Feng, 2021), and perceived functional and emotional value, etc. (Zhao and Wang, 2021); and the response variable R, which mainly includes: consumers' willingness to purchase, persistence, and intention to recommend sharing (Liu et al., 2018; Gao et al., 2019; Wei and Wan, 2020; Guo et al., 2021; Zhao and Feng, 2021).

In general, there is a “black box” between visual marketing and consumers' purchase intention. Most of the existing literature on visual marketing focuses on summarizing the experience and skills of store decoration, which is subjective and has not been upgraded to a universally applicable theoretical result. Drawing on the mature “Stimulus (S)-Organism (O)-Response (R)” model paradigm, combined with empirical analysis, to clarify the mechanism of the impact of visual marketing on consumers' purchase intention, so as to target the visual optimization of stores, enhance store traffic and conversion rate is the significance of this study.

Focusing on visual marketing strategy, in general it is a combination of a series of visual display tools to better present products to consumers and increase their purchase intention in terms of buying scenarios and shopping experience (Morgan et al., 2009; Jadva et al., 2010; Steidle and Werth, 2014; Bertamini et al., 2016; Sevilla and Townsend, 2016; Hussain, 2019; Liu et al., 2019; Gao et al., 2020; Huang et al., 2020). Therefore, how to develop a comprehensive marketing strategy to awaken and stimulate consumers' purchase intention through the stimulation of multi-dimensional visualization in offline stores is a significant issue that fast-fashion apparel brands need to address.

## 3 Theoretical model

**Figure 1:**
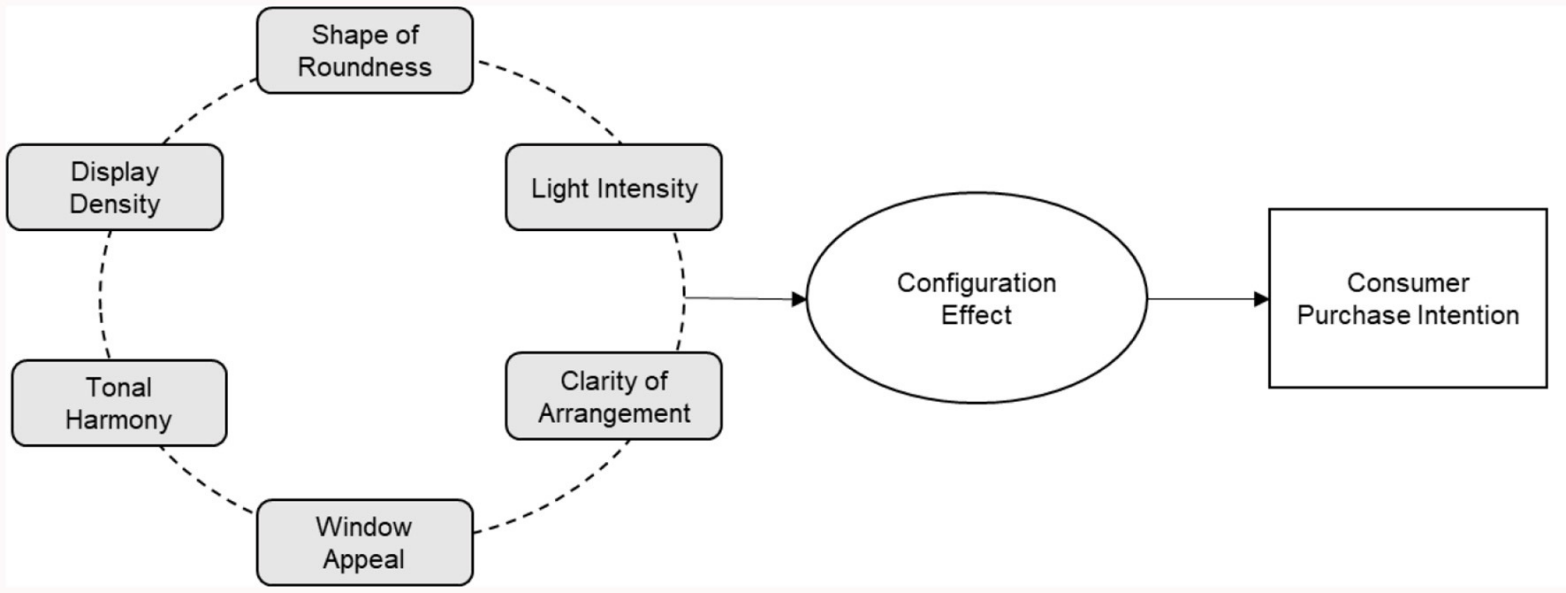
Conceptual model.

According to the above-mentioned literature combing, it is found that there is a wealth of research on visual marketing, especially in the fast fashion industry, which is ahead of other industries in the application of visual marketing. However, most of the research has focused on the single dimension of visual marketing strategy especially display dimension of clothing, for example, existing research indicated that effective display can stimulate consumers' perception of the clothing, thus creating a deeper interaction with the clothing and the brand (Zhu et al., 2018; Han and Xu, 2019; Choi et al., 2020). In addition, several scholars investigated the interaction between apparel display and another visual dimension, and how it influences consumer behavior (M'Hallah and Bouziri, 2016; Hwang et al., 2021; Shi et al., 2021; Li et al., 2022). As mentioned above, there is less research on the impact of visual marketing on consumers' purchase intention through comprehensive dimensional combing, such as display, shape, lighting, and color. By systematically sorting out the dimensions and comprehensively exploring the influence of various factors on consumers' purchase intention from the perspective of visual marketing, this paper can make a contribution to the research on the influence of visual marketing and consumers' purchase intention. In addition, in the field of visual marketing research, there are few studies that use the perspectives on configuration effect to break through the limitations of the traditional methods to study the impact of multi-dimensions of visual marketing on consumers' purchase intention. The configuration effect takes a holistic analytical perspective, viewing the study object as a grouping of different combinations of condition variables (Kumar et al., 2022). It not only integrates the strengths of case studies and variable studies, and discovers the aggregated relationships between elemental groupings and outcomes through ensemble analysis, but also helps to answer causal complexity questions such as multiple concurrent causality, causal asymmetry and multiple scenario equivalence (Du and Jia, 2017). Therefore, it is worthwhile to analyze the impact of visual marketing on the purchase intention of consumers of fast fashion apparel brands through the configuration perspective by using fsQCA method. The conceptual model of this study is showed in Figure 1.

Based on the literature and discussion above, six dimensions of visual marketing were indicated:

1. Shape of roundness (RS) is used to evaluate the line characteristics of the shapes of various types of facilities in offline shops (Jadva et al., 2010; Bertamini et al., 2016). The design of the store is the way in which the brand's emotions are expressed and ideas are expressed. The rounded shape expresses friendly, intimate, warm and soft design feeling, suitable for use in home, children, women, and other supplies, is a very important style trend in design style (Bertamini et al., 2016). Rounded shape is a form of emotional expression, easier to impress consumers.
2. Light intensity (LI) is used to evaluate the lighting in offline shops (Steidle and Werth, 2014). Lighting intensity can affect not only the atmosphere of the space, but also mobilize the customer's mood, stimulate the purchase intention. And good light intensity includes the uniformity of illumination and focus on the proportion and distribution of light, light levels and other issues.
3. Clarity of arrangement (CA) is used to evaluate the overall neatness and clarity of the product arrangement in an offline shop (Ye et al., 2016). Clear arrangement is characterized by simplicity, practicality, product orientation and environmental awareness, and focus on creating a comfortable shopping environment and enhancing the customer shopping experience (Ye et al., 2016). Simple display, multi-level display, emphasis on seasonality and theme, as well as environmental awareness can present customers with an eye-catching shopping experience, making it more convenient for customers to find and select the required products, while highlighting the characteristics and quality of the products, and match the brand's image and philosophy.
4. Display density (DD) is used to evaluate the density of product displays in offline shops (Sevilla and Townsend, 2016). Specifically, the display density of a store is the density of items displayed in a fixed space, and a good display density improves the user experience. Display density is too large, the distance between the products is close, may cause product stains, scratches and other phenomena, affecting the product display effect. On the contrary, if the display density is too small, it may make the products appear to be isolated, causing a cold feeling (Sevilla and Townsend, 2016). Appropriate display density can let the products keep the appropriate distance between the products, not only to ensure the display effect of the products, but also let the consumers can better appreciate and select.
5. Tonal harmony (TH) is used to evaluate the appropriateness of the use of color in offline shops (Babin et al., 2003). The harmony of colors can directly affect the overall appearance of the store and the interior integrity and character. Different color coordination can produce different visual effects. For example, use bright colors in contrast to darker colors, such as black and white, red and black. This color scheme will attract attention and make the store look more eye-catching and prominent. Warmer tones can convey warmth, energy and a sense of welcome. Cold tones can convey a sense of calm, serenity and professionalism. Choose adjacent colors such as blue and purple, red and orange. This color scheme usually creates a sense of calm, harmony and softness. Choose complementary colors, such as red and green, blue and orange. Contrasting colors can create a strong visual impact and make a store more noticeable.
6. Window appeal (WA) is used to evaluate the extent to which an offline shop's window raises consumers' desire to enter the shop (Morgan et al., 2009). Window display is the “face” of the store, a cleverly designed window can attract pedestrians' footsteps in these few seconds, persuading them to enter the store. Window design style and clothing display, is the most intuitive and effective way to push the store and product image to the consumer's line of sight. Store window is not only a part of the overall decoration of the facade, but also the first exhibition hall of the store, it is based on the store's sales of goods, clever use of scenery, props, background screen decoration as a backdrop, with appropriate lighting, color, and text instructions, is a comprehensive advertising art form for the introduction of commodities and product promotion.

## 4 Research design

### 4.1 Research methodology

This study uses a fuzzy set qualitative comparative analysis (fsQCA) approach to explore the complex causal relations by which elements of visual marketing influence consumer purchase intentions. fsQCA adopts a holistic perspective and conducts a cross-case comparative analysis that is dedicated to exploring the causal complexity of which groups of conditional elements cause the emergence of desired outcomes and which groups cause the lack or absence of desired outcomes (Fiss, 2011; Du and Jia, 2017; Pappas and Woodside, 2021; Kumar et al., 2022). The elements of visual marketing will combine to produce different marketing effects. In response to different consumer preferences, different marketing groupings may have diverse and complex effects on consumers' purchase intentions. Secondly, the fsQCA method not only makes up for the shortcomings of qualitative research methods by using a large sample set of cases to address the applicability and uniqueness of traditional qualitative analysis; but also compensates to a certain extent for the shortcomings of large sample analysis for individual phenomenon analysis in quantitative research methods (Kusa et al., 2021; Huang et al., 2022). Finally, this paper focuses on the “joint effect” of the various elements of visual merchandising on consumers' purchase intentions and the “interaction” between different indicators, in order to find the best way to improve the effectiveness of marketing.

### 4.2 Data collection

In fsQCA studies, the representativeness of sampling directly affects the results, and the valid sample equals appropriately selected cases (Du and Jia, 2017). fsQCA is a case-oriented research method which should follow the principles of theoretical sampling and select samples based on the characteristics of the theory and cases (Du and Jia, 2017). Based on the definition and criteria of fast fashion apparel brands in the existing literature, this study selected apparel brands as the subjects of the study which have similar business models, i.e., following fashion trends, launching new products quickly and selling them at lower prices. In the selection of cases of fast fashion clothing brands, this paper follows three conditions from the theoretical and practical levels: firstly, the cases are representative, i.e., the selected case companies have adopted visual marketing measures and have dominated the competition in the industry at a certain stage. Secondly, the case portfolio is differentiated. Companies at different stages of development have their own characteristics in terms of positioning, and when these cases are combined, they will have greater variability and are ideal for cross-case comparative analysis. Thirdly, the data is available. Qualitative comparative analysis requires in-depth analysis of multiple cases, and therefore requires an appropriate amount of case data as research support.

The fast fashion brands selected for this study all have similar business models, i.e., they follow fashion trends, launch new products quickly, and sell them at lower prices. These brands are similarly priced, with most apparel selling for <$500, and inferior in quality to other higher priced clothing brands. In addition, the target customers of the fast fashion brands are similar, mostly younger consumers age from 18 to 40, who have a strong desire for fashionable things but cannot afford the high price of clothing and do not have high requirements for the quality of clothing. However, the visual experience of the offline shops of different brands varies greatly, which also affects consumers' purchase intention from various fast fashion clothing brands. In this study, we obtain first-hand data through experience and observation, and combine it with secondary sources such as relevant papers to conduct a case study of each brand.

The data was obtained by means of a questionnaire sent to consumers, and the case sample was selected to target 15 well-known domestic and international fast fashion brands with a large number of offline shops in China, and five physical shops of each brand in Beijing were selected for data collection. As Beijing is a first-tier city, consumers are more sophisticated and discerning in their aesthetic and shopping experience, and brands focus more on the sensory comfort that visual marketing strategies brings to consumers. These brands include: H&M, UNIQLO, ONLY, VERO MODA, LEDIN, Sanfu Fashion, YISHION, PEACEBIRD, GXG, JNBY, ZARA, HOTWIND, SEMIR, MJstyle. The questionnaires were targeted at consumers who walking around in the physical shops without purchasing behavior. Because this study is to investigate the synergy effect of visual marketing dimensions on consumers' purchase intention, if the sample of this study has already occurred a purchase behavior, it means that these anticipants have already developed a purchase intention, and therefore it will interfere the study and lead to wrong results. In order to ensure that enough valid questionnaires are collected, small gifts were given as a token of appreciation upon completion of the questionnaires to ensure the completeness and validity.

### 4.3 Conceptual measurement

Through combing through the literature, this study has screened and categorized the factors influencing consumers' purchase intention from a visual marketing perspective, and finally identified six indicators.

Shape of roundness (RS) including seats, cabinets, hanging brand logos, etc. Round and oval shapes have an amenity that are more rounded, while polygonal shapes are less rounded (Jadva et al., 2010; Bertamini et al., 2016). Rounded shapes, on the other hand, evoke a feeling of warmth and comfort, while sharp-edged shapes, on the contrary, evoke a feeling of threat. As a result, consumers are more attracted to objects with rounded shapes and more resistant to objects with sharp corners.

Light intensity (LI) is used to evaluate the lighting in offline shops (Steidle and Werth, 2014). At high light intensities, the interior of a shop is brighter, while the opposite is darker. Consumers are more alert and sensible in a brighter environment and are more restrained in their purchases.

Clarity of arrangement (CA) is used to evaluate the overall neatness and clarity of the product arrangement in an offline shop (Ye et al., 2016). A high level of clarity means that the products are well arranged and partitioned, including the placement of models in the shop, which provides a strong guide for consumers to walk and search for products, and allows them to see the products in the shop at a glance. A neatly arranged product will help to reduce the cost of searching for products and is likely to be well received by consumers.

Display density (DD) is used to evaluate the density of product displays in offline shops. Consumers prefer a lower display density (Sevilla and Townsend, 2016). On the one hand, lower display density gives consumers a greater sense of dominance and higher social status; on the other hand, a smaller number of products per unit of space makes consumers aware of the scarcity and preciousness of products.

Tonal harmony (TH) is used to evaluate the appropriateness of the use of color in offline shops (Babin et al., 2003). Cooler shades (e.g., blue, green) are preferred to warmer shades (e.g., red, yellow), as they make consumers feel more comfortable and happier to browse and buy products. In addition to the overall color palette, the use of local color should not be overlooked. For example, consumers' anxiety levels may increase due to the use of warm colors in the checkout area, which may lead to dissatisfaction with the checkout time; the price of an item is usually indicated in black, but if the price is indicated in red, consumers will value the price attribute more and believe they are getting a great deal. In addition, warm colors give consumers a sense of density, while cool colors make them feel sparse. Also, consumers are used to looking from left to right and will feel denser on the left. If the order in which the warm and cold colors are displayed matches the visual habits of the consumer, it will greatly enhance the visual perception of the consumer.

Window appeal (WA) is used to evaluate the extent to which an offline shop's window raises consumers' desire to enter the shop (Morgan et al., 2009). Attractive windows trigger curiosity and increase the rate of entry. It is important that the window displays are a vivid representation of the brand and that they fit in with the overall style of the shop, in order to attract consumers. It is also important to keep the window display fresh by changing the products as sales demand dictates.

In order to ensure the reliability and validity of the scales in this study, the variables were measured using established scales from existing scholars, with reasonable modifications to suit the purpose of this study. Secondly, in order to accurately reflect the variability between cases, the study used a four-value assignment method with a mean distribution, i.e., “0” for no affiliation at all, “0.33” for partial non-affiliation, “0.67” indicates partial affiliation and “1” indicates full affiliation.

In the consumer purchase intention (PI) section, this paper adopts Gunawan and Huarng (2015) study to design a purchase intention scale through three dimensions: demand, repurchase and recommendation. The scale uses a 5-point Likert scale with scores from 1 to 5 indicating a progressive increase in agreement, with scores of 1 and 5 indicating strongly disagree and strongly agree respectively, scores of 2 and 4 indicating disagree and agree respectively, and 3 indicating a neutral attitude (see Table 1).

**Table 1:** Reliability and validity tests of variables.

**Variables**	**Variables measurement question items (basis for assignment)**	**Scale source**	**Crobach's alpha**	**KMO**
Roundness of shape (RS)	Generally rounded shape of the facilities in the shop	Jadva et al., 2010; Bertamini et al., 2016	0.927	0.868
	More of the facilities in the shop are rounded, less of them have sharp corners			
	More of the facilities in the shop have sharp corners, less of them have rounded shapes			
	Almost all the facilities in the shop have sharp corners			
Light intensity (LI)	Overall brightness of the shop is low	Steidle and Werth, 2014	0.826	0.873
	The light inside the shop is soft, the overall brightness is not high			
	Overall brightness in the shop, with some areas darker			
	The overall environment in the shop is very bright			
Clarity of arrangement (CA)	The layout of the store is clear and well organized, with clothing arranged in a clear pattern and neatly arranged. Models or signs listed in the shop provide strong guidance. The clothes are displayed in a variety of directions so that consumers can see the front of the clothes from all angles and can easily find the clothes they need	Ye et al., 2016	0.930	0.921
	Reasonable zoning layout, regular arrangement of clothing, with guidance signs and strong guidance. The clothes are displayed in a variety of directions, making it easy to find the clothes you need			
	The clothing is partitioned and arranged in a certain way, with no or little guidance. Clothes are displayed in a single direction and it still takes time to find the clothes you need			
	No partitioning, clothes are not clearly arranged and appear very disorganized. No mannequins or signage to help guide you or the guidance is not effective. Clothes are displayed in a single direction, making it difficult to find what you are looking for			
Display density (DD)	Adequate aisles are left open and clothing is loosely displayed	Sevilla and Townsend, 2016	0.950	0.869
	Aisles are relatively empty and clothing is loosely displayed			
	Narrow aisles, dense clothing displays			
	Narrow aisles, dense clothing display			
Tonal harmony (TH)	Cool tones overall, reasonable use of local color, order of clothing display in line with consumer visual habits	Babin et al., 2003	0.913	0.854
	Overall cool tones, local color use is reasonable, clothing shades are displayed in a sequence more in line with consumer visual habits			
	Overall warm tones, local use of color is inappropriate, overall warm tones, local use of color is incompatible, clothing shades display order is not in line with the visual habits of consumers			
	Overall warm tones, partial use of color is inappropriate, the order of display of clothing shades does not conform to the visual habits of consumers			
Window appeal (WA)	The window content is very vivid, the window content is always updated	Morgan et al., 2009	0.949	0.912
	The window content is vivid, and the window content is updated more frequently			
	Window content is boring and is updated less frequently			
	Dull or no window, rarely updated			
Purchase intension (PI)	I would like to spend money in the brand's offline shops if I had the need to do so.	Gunawan and Huarng, 2015	0.935	0.827
	I would want to buy clothing from the brand's offline shop more than once			
	I would be happy to recommend the brand's offline shop to others			

### 4.4 Reliability and validity analysis

The reliability test reflects the consistency of the measurement results and the stability of the data, and generally uses Cronbach's alpha value to judge. In this study, the SPSS18.0 software was used to test the reliability of the questionnaire. The results are shown in Table 1, and the Cronbach's alpha values based on the standardized items are all >0.8, indicating that the reliability of this questionnaire is very good (Hair, 2009).

Secondly, validity assesses how close the measurement results are to the intended target and reflects the validity of the scale. The present questionnaire refers to established scales that are available and are better in terms of content validity. In addition, the KMO and Bartlett's sphericality values were calculated for each variable, where the KMO and factor loading coefficients were >0.7 and the Bartlett's sphericality values were significant. Secondly, the fit between the study variables and the measured items was tested using AMOS 24.0, where CMIN /df = 1.79, standard fit index (NFI) = 0 0.94, goodness of fit index (GFI) = 0.92, comparative fit index (CFI) = 0 0.95, Tucker-Lewis index (TLI) = 0 0.94, adjusted goodness of fit index (AGFI) = 0.89, RMSEA = 0.017. Therefore, the validity of the questionnaire was good.

## 5 Research results and discussion

### 5.1 Data description

A total of 473 questionnaires were distributed in this study, of which 435 valid questionnaires were obtained after removing invalid questionnaires such as missing answers and inconsistencies, with an effective rate of 91%. The results of the descriptive statistical analysis show that among the 435 valid questionnaires, there were 326 female consumers, accounting for 75%, which shows that the target consumers of fast fashion clothing brands are mostly female. The results of this study show that the young and middle-income consumers prefer fast fashion brands due to their low prices, quick updates and trendiness. The data for each dimension of consumers' willingness to buy and the mean values were calculated and used as the final indicator data for the outcome variable.

### 5.2 Calibration of conditional variables

Before conducting the qualitative comparative analysis of fuzzy sets, the variables need to be calibrated for fuzzy sets, i.e. three calibration anchor points are set: fully affiliated, crossover, and fully unaffiliated, according to the relevant theoretical knowledge and data situation, assigning each case a set affiliation distributed between 0 and 1 (Du and Jia, 2017; Pappas and Woodside, 2021). For the calibration of the independent variables, a direct calibration was chosen, assigning values of 0, 0.22, 0.67, and 1 to the different cases, respectively fully affiliated, “3” is the crossover point and “2” is fully unaffiliated (Du and Jia, 2017; Pappas and Woodside, 2021). The calibration results for each variable are shown in Table 2.

**Table 2:** Results of variables calibration.

**Brand**	**RS**	**LI**	**CA**	**ID**	**TH**	**WA**	**PI**
H&M	0	0.67	0.67	0.33	1	1	0.87
UNIQLO	0.33	0	0.67	1	1	1	0.93
ONLY	0.67	0.67	0.67	0.67	0.33	0	0.79
VERO MODA	1	0.33	0.67	1	0.33	0.67	0.73
LEDIN	1	0.67	0.67	0.67	0.33	0.33	0.77
Sanfu	1	1	0.33	0	0	1	0.57
YISHION	0.33	0.67	1	0.67	0.67	1	0.62
Peace Bird	0.33	0.33	0.67	1	0.33	0.33	0.67
GXG	0	0	0.33	0.67	1	0	0.47
BSIJA	0.33	0.67	0	0.67	0.33	0.33	0.32
JNBY	0	0.33	0.33	0.33	0.33	0.33	0.5
ZARA	0.67	0.67	1	1	1	1	0.9
Hotwind	0.33	1	1	0.33	1	0.67	0.66
Semir	0	0.67	0.67	0.33	0.67	0.67	0.58
MJstyle	0.33	0.67	0.67	0.33	1	0.33	0.56

### 5.3 Necessary conditions analysis

Since the necessary conditions may be simplified in the group analysis process, this study starts with a necessary condition analysis for a single condition (Fiss, 2011; Pappas and Woodside, 2021). In fsQCA, a “necessary condition” means that the condition is always present when the result is present; if the condition is not present, the result cannot be generated. In general, the antecedent condition is considered necessary for the outcome variable when the consistency is > 0.9 or close to 0.9 (Douglas et al., 2020; Pappas and Woodside, 2021; Kang and Shao, 2023). The results of the consistency analysis of the necessary conditions for high/non-high consumer purchase intention are shown in Table 3. The consistency of all the antecedent conditions in this study was < 0.9, which indicates that none of the antecedent variables in this paper were necessary for high/non-high consumer purchase intention. This also suggests that the impact of visual merchandising on consumer purchase intention is complex and is the result of a combination of variables that cannot be explained by one variable alone and requires further analysis of the variables, i.e., group analysis.

**Table 3:** Consistency analysis of necessary condition.

**Conditional variables**	**Resulting variables**	
	**High consumer purchase intention**	**Non-high consumer purchase intention**
RS	0.541	0.585
~RS	0.662	0.814
LI	0.702	0.846
~LI	0.590	0.729
CA	0.838	0.773
~CA	0.453	0.798
ID	0.768	0.838
~ID	0.521	0.729
TH	0.761	0.812
~TH	0.476	0.652
WA	0.748	0.729
~WA	0.500	0.759

On the basis of the truth table generated, the fsQCA 3.0 software was used to carry out the standard analysis and three types of solutions were obtained: complex solutions that do not use logical residuals, intermediate solutions that use reasonable logical residuals, and simple solutions that use all logical residuals (see Table 4). The intermediate solution should be the main one as the process of generating the intermediate solution is more reasonable and does not simplify the necessary conditions. On this basis, the parsimonious solution is used as a basis for distinguishing between core and marginal conditions. Specifically, antecedent conditions that occur only in the intermediate solution are marginal conditions, while core conditions will exist in both the simple and intermediate solutions.

**Table 4:** Truth table of non-high consumer purchase intention.

**RS**	**LI**	**CA**	**ID**	**TH**	**WA**	**Number**	**~PI**	**Raw consist**.	**PRI consist**.	**SYM** **consist**
0	1	0	1	0	0	1	1	0.940	0.745	0.745
0	0	0	1	1	0	1	1	0.902	0.212	0.368
0	0	0	0	0	0	1	1	0.825	0.033	0.077
0	1	1	0	1	0	1	1	0.824	0.000	0.000
0	1	1	1	1	1	1	0	0.758	0.000	0.000
1	1	0	0	0	1	1	0	0.744	0.029	0.040
1	1	1	1	1	1	1	0	0.725	0.000	0.000
0	0	1	1	0	0	1	0	0.723	0.000	0.000
1	0	1	1	0	1	1	0	0.699	0.000	0.000
0	0	1	1	1	1	1	0	0.654	0.000	0.000
0	1	1	0	1	1	3	0	0.627	0.000	0.000
1	1	1	1	0	0	2	0	0.520	0.000	0.000

The results of the software analysis are shown in Table 5, in which there are 2 groups that produce high consumer purchase intention, and the consistency index of both groups is >0.95, which is very high, and these 2 groups are sufficient conditions to produce high consumer purchase intention. The coverage of the solution is 0.782, indicating that these 2 groups explain the main reasons for high consumer purchase intention. In contrast, there are 2 groupings that lead to non-high consumer purchase intentions, with an overall consistency index of 0.824, which has a high consistency. The coverage of the solution was 0.759, explaining most of the reasons for the non-high consumer purchase intention.

**Table 5:** Configuration of consumer purchase intention.

**Conditional variables**	**High consumer purchase intention**		**Non-high consumer purchase intention**	
	**H1**	**H2**	**NH1**	**NH2**
RS				
LI		•		
CA	•	•		◇
ID	•		◇	
TH		•		
WA		•	◇	◇
Consistency	0.979	0.970	0.813	0.852
Coverage	0.689	0.488	0.534	0.672
Unique coverage	0.294	0.093	0.087	0.225
Solution consistency	0.969		0.824	
Solution coverage	0.782		0.759	

•Indicates the presence of a core causal condition, • indicates the presence of a marginal causal condition,◇ indicates the absence of a core causal condition,◆indicates the absence of a marginal causal condition, and “blank” indicates that the presence or absence of the condition does not affect the outcome.

## 6 Research results and analysis

The study investigated the paths and groupings of visual marketing on consumers' purchase intentions through the fsQCA analysis method. With the complex effect of seven visual factors: shape roundness, light intensity, arrangement clarity, display density, color coordination and window attractiveness, the study reached the following conclusions: (1) There are multiple conditional groupings of visual marketing on the paths of consumers' purchase intentions; (2) Two paths were found to match the grouping of high consumer purchase intentions. (3) There is a causal asymmetric relationship with the driving mechanism of consumers' purchase intention; (4) All four histolytic paths of high/non-high consumer purchase intention have good coverage and consistency, and the data and results can well explain the research questions in this paper, with the specific histolytic paths explained as follows.

### 6.1 Analysis of the generation mechanism of high consumer purchase intention

H1: CA^*^ID, the path shows that regardless of whether the offline shops have better shape roundness, light intensity, color coordination and window appeal, as long as the clothes are neatly arranged and clear, the display is not too crowded and there is a more spacious environment, consumers will have a higher willingness to buy. The reason for this may be that in the process of laying out the shop and designing the visual merchandising effect, the higher clarity and lower density of the display will create a sense of comfort and reduce the time it takes for consumers to lock in their clothes, giving them an impression of the brand as reliable and premium, and thus increasing their willingness to buy. Compared to foreign fast fashion brands that have developed maturely, domestic fast fashion brands still lack talent in offline shop display design, and elements such as the artistry of shop visuals still have some gaps with foreign brands, but doing a good job of clearly arranging clothing and preventing cramped and crowded shops can still rise above the siege of many foreign fast fashion brands and gain consumer recognition. Among the 15 case studies in this study, domestic brands LEDIN and Echon are typically successful in terms of shelf product arrangement and display.

H2: LI^*^CA^*^TH^*^WA, the path shows that when designing and marketing the visual image of an offline shop, even if there is no advantage in terms of roundness of shape and density of display, as long as the arrangement is neat and clear, the color palette is coordinated, the lighting is soft and an attractive window is configured, consumers can generate a high willingness to buy. It is worth noting that clarity of arrangement is found in both the first and second paths, suggesting that a neat and clear arrangement is essential to a good visual experience in a physical shop. For fast fashion brands with limited space in their shops, even if they cannot guarantee a low density of displays, they can still gain consumer acceptance through appropriate light intensity, high tonal harmony and window appeal, but only if they have a high level of clarity.

In addition, the data in Table 5 shows that the H1 path has higher coverage than the H2 path. This result indicates that in the 15 brand cases selected for this study, most fast fashion apparel brands achieved high consumer purchase intentions through the first path. This further validates the importance of the grouping paths between arrangement clarity and display density on consumer purchase intention. The two different groupings also reflect the diversity of paths to high consumer purchase intent. Light intensity, tonal harmony and window appeal also influence consumer purchase intentions. Shape roundness does not appear in either path, suggesting that shape as an indicator may have a low impact on consumer purchase intentions.

### 6.2 Analysis of the generation mechanism of non-high consumer purchase intention

Du and Jia (2017) proposed that the QCA approach is asymmetrical, so the causes that generate high consumer purchase intention are not necessarily the reverse causes that lead to non-high purchase intention. In order to fully explore the impact of visual marketing dimensions on consumer purchase intention, this paper also analyses the groupings that lead to non-high purchase intention as follows.

NH1: ~ID^*^~WA, this path suggests that apparel brands that have neither a more reasonable display density nor a higher window appeal are unable to achieve high consumer purchase intentions even if they have more reasonable elements such as lighting and color tones. The reason for this phenomenon may be due to the lack of a strong attractive shop window, it is difficult to attract the attention of consumers, there is no greater incentive to enter the shop, coupled with excessive density of clothing display, consumers feel crowded and uncomfortable, thus reducing the desire to spend.

NH2: ~CA^*^~WA, this path illustrates that if the offline shop windows of clothing brands are boring and the apparel arrangement is confusing, it will lead to lower consumer willingness to purchase. In contrast to the first path, not having window appeal is a cause of lower purchase intentions. This shows the importance of the dimension of window appeal on consumers' purchase intention.

In addition, the coverage of the grouping NH1 is slightly lower than that of the grouping NH2 with 53% and 67% respectively, i.e., it is mainly these two groupings that reduce consumers' purchase intention. A comprehensive analysis of the results for each grouping shows that arrangement clarity, display density and window appeal are the elements that have a greater impact on consumers' purchase intention.

### 6.3 Robustness analysis

Checking the robustness of the analysis results is a key step in QCA studies (Greckhamer et al., 2018). In this study, the data were analyzed again after adjusting the case frequency to 2 and the consistency threshold to 0.81 to compare the changes in the groupings to assess the results. It was found that the combination of paths affecting consumers' purchase intentions did not lead to substantial changes in the number of groupings, their components, or their consistency and coverage after the parameters were adjusted. Therefore, it was concluded that the results of the analysis obtained in this study were reliable and robust (Greckhamer et al., 2018).

## 7 Conclusion

### 7.1 Research findings

From a consumer perspective, the position of “beauty” in the user value chain has seen a rapid jump (Jinyoung Yoo et al., 2023). Along with the development of industrial design and user experience science, the supply of “beauty” has increased dramatically, and consumers are clearly willing to pay for products that match their visual, experiential and lifestyle ideals. According to previous research, 64% of respondents would buy products that are more visually appealing (Jinyoung Yoo et al., 2023). Therefore, if brands want to increase consumers' purchase intention, visual marketing is the wedge, the best entry point (Barnes and Lea-Greenwood, 2010). In this paper, through a case study of 15 fast fashion apparel brands, we apply the fuzzy set qualitative comparative analysis method to explore the comprehensive effect of shape, lighting and other dimensions on consumer purchase intention from multiple dimensions of visual marketing. The main conclusions are as follows: (1) there are two driving paths for high consumers' purchase intention. The first path is a combination of high clarity of arrangement and low display density; the second path is a combination of low light intensity, high clarity of arrangement, high tonal harmony and high window appeal. (2) There are also two paths that drive non-high consumers' purchase intentions, and they are asymmetrically related to the paths that drive high consumers' purchase intentions. The first path is for the group with high display density and low window appeal, and the second path is for the group with low arrangement clarity and low window appeal; (3) Arrangement clarity, display density and window appeal are the core conditions that have a greater impact on purchase intentions. Light intensity and tonal harmony are marginal conditions and have an average impact on purchase intentions, while roundness of shape is less frequent and less important in each path. Similar to previous studies by scholars, by using visual marketing strategy, fast fashion brands can significantly reduce the cost and increase the attractiveness of consumer. Furthermore, the combination of the 'fast on-trend' nature of fast fashion brands and good visual displays can create a synergy that can increase consumers' purchase intention (Liu et al., 2019; Huang et al., 2020; Chae et al., 2021; Jinyoung Yoo et al., 2023).

### 7.2 Theoretical implications

Visual marketing refers to the means of communicating brand concepts and brand marketing through visual aids (Zhang and Zhang, 2023). A good marketing strategy cannot be achieved without visual aids. Existing research shows that people can't communicate without visuals for the most part, and that visual content is 40 times more likely to be shared than other forms of content (Zhang and Zhang, 2023). This is because visual content is often more engaging, easier to understand, and creates longer lasting memories than text alone. Visual marketing can turn intangible information into tangible, rich images, leaving a deeper impression of your brand through visual content. Compared with existing studies related to visual marketing of fast fashion brand, this paper has the following theoretical contributions.

Firstly, by combing the existing related literature and based on SOR theory, for the first time, this paper selects the important dimensions that affect the consumers' purchase intention of fast fashion brand, constructs a new and multi-dimensional variable model, put the six dimensions including shape of roundness, light intensity, clarity of arrangement, window appeal, tonal harmony and display density into the same research system and discovers the histological conditions for high/non-high consumers' purchase intention generation, providing a new approach and research perspective for fast fashion brands, visual marketing and consumer research.

Secondly, based on the existing research, this paper enriches the research related to visual marketing strategy of fast fashion brand, especially for the offline shore, as existing research mainly focusing on how to build the visual marketing strategy of online shop and ignore the significant of real shopping experience of consumers. The results of the study reveal that under the intense industry competition, how the multi-dimensional visual marketing combination strategy adopted by fast fashion brand in the offline store can improve consumer's purchase intention and marketing performance, and the specific causal relationship between them, which further enriches the research related to the visual marketing strategy.

Finally, most of the existing literature examines the net effect of a single variable on consumer purchase intentions from the perspective of a single variable, with fewer studies focusing on the combined effect of a complex grouping of multiple antecedent variables on consumer purchase intentions. Therefore, this study uses the QCA research method, combining the advantages of qualitative and quantitative research to provide new research methods and ideas on complex causal relationships, and has some theoretical implications for the marketing and development of fast fashion clothing brands.

### 7.3 Management implications

The findings of this study provide a new focus and direction for the visual marketing strategies of fast fashion clothing brands.

Firstly, the display of clothing is the first impression that a brand leaves on consumers. A reasonable, neat and beautiful display of clothing can better show the color, form and vitality of clothes, which is an important influencing factor for consumers to select and buy products in the shop. Fast fashion clothing brands should pay extra attention to clothing display, and should ensure that the clothing is neatly arranged on the premise of the implementation of other visual marketing methods, such as: model display, scientific classification, decorative backdrop, etc. In addition, brick-and-mortar shops need to arrange for specialized staff to sort and categories clothing in offline shops, and to restore hangers that are more disorganized after consumers have taken them.

Secondly, the lighting design of fast fashion shops needs to be balanced from the outside to the inside. While brands communicate their ideas through light intensity and color, it is most important to design an overall lighting atmosphere that makes customers feel comfortable and have a desire to spend. Fast fashion shops should pay attention to the visual optimization of the overall environment, avoiding excessive light intensity and using softer lighting as much as possible to create a good shopping atmosphere. In terms of color usage, try to use gradual cold tones that can bring a sense of relaxation and cheerfulness to consumers. Secondly, attention should be paid to the appropriate use of warm and cold colors in different areas, for example, it is not advisable to use warm colors in the checkout area that enhance consumer anxiety.

Once again, the window is the most intuitive first visual impression of the brand to consumers, showing the brand's style, positioning, product type, etc. It is an important element to attract consumers into the shop. Fast fashion clothing brands should reasonably design the overall style of their physical shop windows to reflect the characteristics of the brand, and update the windows in time for different seasons and festivals to ensure that the contents of the windows fit with the sales nodes. The attractiveness of the windows can be enhanced through the use of models in various poses, props and multimedia to avoid monotonous and boring window content, so as to promote consumers to shop.

Finally, fast fashion shops should try to maintain a more spacious environment to avoid the psychological pressure caused by too much clothing piling up on consumers. For clothing shops with insufficient display space, the use of light, color and windows should be used to reduce the negative impact of high display density on consumers' purchase intention.

### 7.4 Research limitations and future research

This study has some limitations in analyzing the impact of visual marketing on consumers' purchase intention, specifically: firstly, this study focuses on the visual marketing of offline shops of fast-fashion clothing brands, and does not involve much in the current fast-growing e-commerce platforms, which is an important direction for future visual marketing research. Secondly, consumers' senses include not only sight, but also hearing, smell, taste and touch, usually five senses interacting together to influence consumers. Therefore, exploring the complex mechanisms of multiple sensory interactions can also contribute to the study of visual marketing and consumer purchase intentions. Again, due to economic, cultural, aesthetic and technological influences, there are many differences in the positioning, style and design of Chinese and foreign fast fashion clothing brands, and therefore, in future studies, further research can be conducted on consumer purchase intention with regard to the unique characteristics of fast fashion brands in different countries.

## Data availability statement

The original contributions presented in the study are included in the article/supplementary material, further inquiries can be directed to the corresponding author.

## Ethics statement

The studies involving humans were approved by the China University of Political Science and Law. The studies were conducted in accordance with the local legislation and institutional requirements. The participants provided their written informed consent to participate in this study.

## Author contributions

YZ: major of works. SH: data analysis. All authors contributed to the article and approved the submitted version.
